# “I would love if there was a young woman to encourage us, to ease our anxiety which we would have if we were alone”: Adapting the Mothers2Mothers Mentor Mother Model for adolescent mothers living with HIV in Malawi

**DOI:** 10.1371/journal.pone.0217693

**Published:** 2019-06-07

**Authors:** Nicole B. Carbone, Joseph Njala, Debra J. Jackson, Michael T. Eliya, Chileshe Chilangwa, Jennifer Tseka, Tasila Zulu, Jacqueline R. Chinkonde, Judith Sherman, Chifundo Zimba, Innocent A. Mofolo, Michael E. Herce

**Affiliations:** 1 University of North Carolina Project/Malawi, Lilongwe, Malawi; 2 UNICEF/New York, New York, New York, United States of America; 3 School of Public Health, University of the Western Cape, Cape Town, South Africa; 4 Department of HIV and AIDS, Ministry of Health, Government of the Republic of Malawi, Lilongwe, Malawi; 5 Mothers2Mothers/Malawi, Lilongwe, Malawi; 6 UNICEF/Malawi, Lilongwe, Malawi; 7 Institute for Global Health & Infectious Diseases, Department of Medicine, University of North Carolina School of Medicine, Chapel Hill, North Carolina, United States of America; USC Keck School of Medicine, Institute for Global Health, UNITED STATES

## Abstract

**Background:**

Pregnant and post-partum adolescent girls and young women (AGYW) living with HIV in sub-Saharan Africa experience inferior outcomes along the prevention of mother-to-child transmission of HIV (PMTCT) cascade compared to their adult counterparts. Yet, despite this inequality in outcomes, scarce data from the region describe AGYW perspectives to inform adolescent-sensitive PMTCT programming. In this paper, we report findings from formative implementation research examining barriers to, and facilitators of, PMTCT care for HIV-infected AGYW in Malawi, and explore strategies for adapting the mothers2mothers (m2m) Mentor Mother Model to better meet AGYW service delivery-related needs and preferences.

**Methods:**

Qualitative researchers conducted 16 focus group discussions (FGDs) in 4 Malawi districts with HIV-infected adolescent mothers ages 15–19 years categorized into two groups: 1) those who had experience with m2m programming (8 FGDs, n = 38); and 2) those who did not (8 FGDs, n = 34). FGD data were analyzed using thematic analysis to assess major and minor themes and to compare findings between groups.

**Results:**

Median participant age was 17 years (interquartile range: 2 years). Poverty, stigma, food insecurity, lack of transport, and absence of psychosocial support were crosscutting barriers to PMTCT engagement. While most participants highlighted resilience and self-efficacy as motivating factors to remain in care to protect their own health and that of their children, they also indicated a desire for tailored, age-appropriate services. FGD participants indicated preference for support services delivered by adolescent HIV-infected mentor mothers who have successfully navigated the PMTCT cascade themselves.

**Conclusions:**

HIV-infected adolescent mothers expressed a preference for peer-led, non-judgmental PMTCT support services that bridge communities and facilities to pragmatically address barriers of stigma, poverty, health system complexity, and food insecurity. Future research should evaluate implementation and health outcomes for adolescent mentor mother services featuring these and other client-centered attributes, such as provision of livelihood assistance and peer-led psychosocial support.

## Introduction

The rate of adolescent pregnancy in sub-Saharan Africa (SSA) is much higher compared to other regions in the world, with a birth rate among adolescent girls (15–19 years) over 200 births per 1,000 girls [1]. Adolescent girls also experience worse pregnancy and health outcomes compared to adult mothers. Available evidence indicates that adolescent girls have higher risk of preterm delivery, eclampsia, stillbirth, and maternal mortality, and their infants are more likely to have low birth weight and neonatal complications [2–4]. Additionally, many girls who become pregnant during adolescence drop out of, or never enter, secondary school, which limits both their educational and economic opportunities and may further perpetuate poverty and gender inequality [5].

In Malawi, 29% of adolescent girls ages 15–19 years are either pregnant or have one or more children [6]. Adolescent girls from rural areas and the lowest socioeconomic strata in Malawi have the highest pregnancy rates [6]. Specifically, 44% of adolescent girls in the poorest wealth quintile experienced a pregnancy during adolescence compared to 15% of adolescent girls in the wealthiest quintile [6]. Qualitative data from one district in Malawi suggests that higher adolescent pregnancy rates are associated with less educational attainment, lower socioeconomic status, and limited knowledge of reproductive and sexual health [7].

Many of the same social and structural risk factors influencing adolescent pregnancy also increase the risk of HIV acquisition among adolescent girls and young women (AGYW) ages 15 to 24 years. As a result, AGYW account for about 74% of all new HIV infections among adolescent males and females in SSA [8]. While efforts to reach UNAIDS 90/90/90 targets have produced several notable successes, recent data from SSA suggest that AGYW are being left behind [9–11]. For example, in a 2015–2016 assessment in Malawi, 79.6% of all HIV-infected AGYW on anti-retroviral therapy (ART) were virally suppressed compared to 94.1% of HIV-infected women ages 25–34 years [12]. While substantial progress has been made toward HIV epidemic control in Malawi, these data suggest additional efforts are needed to better engage AGYW in the full continuum of HIV prevention, treatment, and care [12–13].

Among pregnant and post-partum AGYW living with HIV in SSA, maternal health care utilization, uptake of prevention of mother-to-child transmission of HIV (PMTCT) services, and HIV care retention are all lower compared to their adult counterparts. Among mother-infant pairs of varying ages presenting for PMTCT services at 120 health facilities in Kenya, HIV-infected adolescent mothers were significantly less likely to attend four or more antenatal visits, be on ART, or have their infants receive anti-retroviral (ARV) prophylaxis than adult HIV-infected mothers [14]. Results from Zimbabwe echo these findings, showing that ART initiation rates were lower among HIV-infected pregnant adolescents (15–19 years) compared to adults [15]. Similarly, a cohort study in Cape Town found that younger maternal age was associated with lower ARV uptake during the antenatal period, less frequent receipt of early infant diagnosis (EID) testing, and higher rates of mother-to-child transmission of HIV (MTCT) [4]. In a facility-based survey from South Africa, HIV-infected adolescent mothers were three times less likely to utilize PMTCT interventions in comparison to adult mothers, resulting in higher rates of MTCT [16]. Indeed, adolescent maternal age has been shown to be one of the main risk factors associated with dropout from the PMTCT cascade [17–21].

Based on these findings, it is clear that current PMTCT and associated support programs in SSA are not adequately meeting the needs of adolescent mothers living with HIV. While PMTCT support programs involving community health workers, mentor mothers, and other task-sharing cadres aim to support all mothers living with HIV, many do not offer services specifically tailored to the unique needs, preferences, and barriers experienced by adolescent mothers. For example, the Mentor Mother Model pioneered by the international non-governmental organization (NGO), mothers2mothers (m2m), employs women living with HIV, who have successfully navigated the PMTCT cascade, to ensure that other mothers living with HIV are engaged with, linked to, and retained in care [22]. Mentor mothers have helped achieve marked MTCT reductions in m2m programs in SSA, with an average MTCT rate of 1.6% documented among m2m clients [23]. Despite the apparent success of the m2m program, however, routine data from 6 SSA countries, including Malawi, demonstrate a gap in outcomes between adolescent and older m2m clients, with the odds of adolescent mothers initiating their HIV-exposed infants on cotrimoxazole prophylaxis and ensuring their infants receive EID testing being lower compared to mothers 25 years of age and older [24].

Thus, existing PMTCT programs must be adapted to address the unique experiences of adolescent mothers living with HIV to overcome the challenges leading to low PMTCT service utilization and inferior health outcomes seen in this population [25–26]. Indeed, evidence-based interventions focused on adolescent mothers are urgently needed to improve their access to comprehensive HIV treatment and care, and to decrease the risk of HIV transmission to their infants and young children [18]. In order to support adaptation of the m2m model to better serve adolescent mothers living with HIV in Malawi’s national PMTCT program, we conducted formative qualitative implementation research to understand this population’s service delivery-related needs, preferences, and experiences. We report our findings here according to established qualitative research criteria with the aim of informing programmatic efforts to increase engagement and retention of pregnant and post-partum adolescent mothers living with HIV along the entire PMTCT cascade [27].

## Methods

### Study setting, design, and sampling

Four districts in Malawi were purposively selected to reflect the geographic, sociodemographic, and epidemiological diversity of the HIV epidemic in Malawi: Mzimba North (Northern Region), Lilongwe (Central Region), Thyolo (Southern Region), and Mangochi (Southern Region). Based on available estimates, HIV prevalence among reproductive-aged women 15 to 49 years varied by study district at 3.9% in Mzimba, 7.9% in Lilongwe, 12.4% in Thyolo, and 13.2% in Mangochi [6]. Within each district, Ministry of Health (MOH) health zones containing at least 2 health facilities were purposively selected to ensure inclusion of at least 1 facility with active m2m programming and at least 1 facility with no m2m programming at the time of data collection.

To capture a diversity of adolescent mother perspectives on PMTCT services in Malawi, the study team worked with healthcare workers (HCWs) and m2m staff based at the selected health facilities in each health zone to recruit a purposive sample of participants from two different beneficiary groups: 1) adolescent mothers living with HIV who had previous experience with the m2m program (i.e. “m2m group”); and 2) adolescent mothers living with HIV who did not have any exposure to the m2m program (i.e. “non-m2m group”). HCWs and m2m staff contacted potential participants and referred them to the study team to learn more about the FGDs, and to complete study screening and consent procedures, if interested. Coordinating with local staff in this way helped ensure that each participant was correctly assigned to the appropriate m2m or non-m2m FGD group, thereby minimizing exposure misclassification. All mothers had received, or were currently receiving, PMTCT services from the MOH national program. At one health zone in Thyolo district, the m2m program had recently stopped programming at one facility; however, the researchers were still able to recruit participants who had previously been enrolled in the m2m program within that health zone.

### Study population

To be eligible for FGD participation, individuals had to: 1) have documented HIV infection; 2) be between 15 and 19 years of age (the age of emancipation in Malawi is 15 years) [28]; and 3) be currently pregnant, or post-partum and/or breastfeeding (out to 2 years post-delivery).

Each focus group included a minimum of three and a maximum of eight participants. Two FGDs were conducted in each of the 2 health zones per study district, for a total of 4 FGDs per district and 16 FGDs overall. Eight of these FGDs were conducted with the m2m group, and the other 8 with the non-m2m group.

### Ethical considerations

The National Health Sciences Research Committee of Malawi (#17/07/1867) and the Biomedical Institutional Review Board of the University of North Carolina at Chapel Hill, United States of America (#17–1908) reviewed and approved the study protocol. All study procedures were conducted in strict compliance with United States and Malawian ethical standards regarding research involving human participants, and all data collection followed international guidelines for Good Clinical Practice. All participants provided written informed consent. The age of emancipation in Malawi is 15 years, and, as such, adolescent potential participants could provide written informed consent without parent or guardian approval from the age of 15 [29]. For illiterate participants, the research team engaged an impartial third party witness unaffiliated with the study to advocate for, and co-sign the consent form on behalf of, the participant.

### Data collection

Two qualitative researchers from the University of North Carolina Project—Malawi, who were trained in qualitative data collection techniques, conducted 16 focus group discussions (FGDs) with consenting pregnant and post-partum/ breastfeeding adolescents living with HIV from September to November 2017.

All data collection tools were developed in English, translated into the three languages commonly spoken in the study districts (i.e. Chichewa, Tumbuka, and Yawo), and then back-translated for accuracy. All English and translated versions of the study tools were approved by all regulatory bodies. A trained moderator and a trained note taker led each FGD using a semi-structured FGD guide (S1 File), which researchers developed using a framework approach to elicit information about the beliefs, perceptions, behaviors, and environmental structures that may enhance or limit PMTCT program effectiveness [30–31]. Specifically the FGD guide explored the following areas of inquiry: 1) HIV-infected adolescent mothers’ beliefs, perceptions, social norms and behaviors; 2) HIV-infected adolescent mothers’ perceived needs for, and barriers to, PMTCT care and social support; and 3) HIV-infected adolescent mothers’ preferences for the types and characteristics of mentor mothers, and location for program encounters (i.e. in the home, clinic, and/or community). The researchers utilized an approach rooted in health systems research and health program quality improvement methodologies to conduct the FGDs [30].

FGDs lasted approximately 2 hours in most cases. All FGDs were conducted in private, secure rooms at MOH health facilities, and there were no non-participants present in the FGD rooms. The FGD moderator and note taker were both females, and they established rapport with the FGD participants during the screening and enrolment process. After obtaining written informed consent, but before beginning FGDs, the research team collected basic socio-demographic information on all participants using a simple case reporting form. All focus groups were audio-recorded. The moderator predominately asked all FGD questions from the guide, as well as all follow-up and clarification questions. The note taker assisted her and maintained detailed field notes during the FGD. She utilized those notes to clarify any discrepancies in the transcripts.

Following the FGDs, all audio files were translated and transcribed by trained qualitative researchers. The transcripts then underwent quality control review by the FGD moderator, FGD note taker, and the first (NBC) and second (JN) authors. During the quality control process, data saturation was discussed. Data saturation was determined through interim review of transcripts, data matrices, and coding memos. Due to the logistical difficulties in reaching and supporting the transport of participants, especially in rural study districts, FGD transcripts were not returned to participants for comment.

### Data analysis

**Fig 1:**
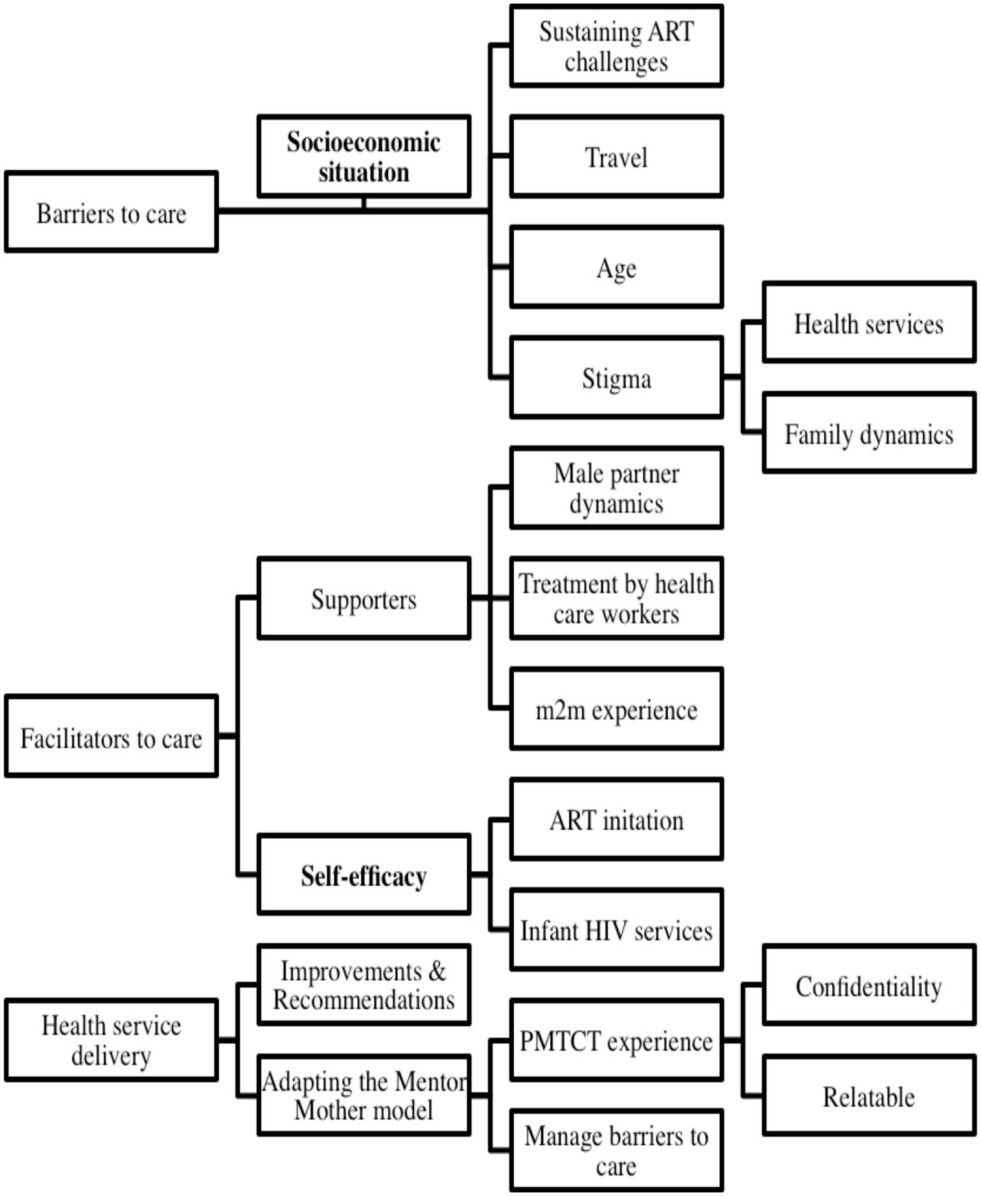
Focus group discussion coding tree.

All FGD transcripts were coded by authors NBC and MEH, both of whom were trained and experienced in qualitative research methods. The coding team first developed a codebook containing only deductive codes based on FGD guide questions. After an initial round of coding, the codebook was updated to incorporate inductive codes identified during the coding process. By the end of FGD coding, the codebook contained a total of 32 deductive and inductive codes. After coding, the researchers used Dedoose Version 7.6.23 (SocioCultural Consults, LLC) to develop a coding tree (Fig 1), and conducted thematic analysis. They also developed data matrices in Microsoft Excel to assess major and minor themes, make comparisons between the m2m and non-m2m FGDs, and identify powerful, illustrative quotes.

## Results

### Participant characteristics

Seventy-two adolescent girls living with HIV participated in the FGDs. None of the eligible potential participants refused participation or withdrew from the study. Participant ages ranged from 15 to 19 years, with the median participant age being 17 years (Table 1). The majority of participants reported that they were married (57%), and 17 participants were never married (24%). The majority of FGD participants were post-partum and breastfeeding (47%, n = 34). Of 38 participants with prior m2m experience, 42% (n = 16) reported previously interacting with m2m Mentor Mothers 10 or more times.

**Table 1:** Demographics of focus group discussion participants.

Variable		All participants (N = 72) % (n)	m2m^a^ (N = 38) % (n)	Non-m2m (N = 34) % (n)
***District***	*Lilongwe*	29% (21)	32% (12)	26% (9)
	*Mzimba North*	22% (16)	24% (9)	21% (7)
	*Mangochi*	25% (18)	24% (9)	26% (9)
	*Thyolo*	24% (17)	21% (8)	26% (9)
***Median age*, *years (interquartile range)***		17 (2 years)	18 (1.75 years)	18.5 (1 year)
***Marital status***	*Married*	57% (41)	58% (22)	56% (19)
	*Widowed*	1% (1)	0% (0)	3% (1)
	*Divorced/Separated*	18% (13)	11% (4)	26% (9)
	*Never married*	24% (17)	32% (12)	15% (5)
***Pregnancy status***	*Pregnant*	32% (23)	37% (14)	26% (9)
	*Post-partum and Breastfeeding*	47% (34)	50% (19)	44% (15)
	*Post-partum*, *not breastfeeding*	21% (15)	13% (5)	29% (10)
***Number of interactions with m2m Mentor Mothers***	*1–2 times*		32% (12)	--
	*3–4 times*		18% (7)	--
	*5–9 times*		8% (3)	--
	*≥10 times*		42% (16)	--

^a^m2m = mothers2mothers

### Barriers to PMTCT care and treatment for adolescent mothers living with HIV

Across all focus group discussions, participants discussed barriers that adversely impacted their uptake of antenatal and HIV health services, retention along the PMTCT cascade, and livelihood as adolescent girls living with HIV. The most frequently mentioned overarching barrier across both m2m and non-m2m FGD groups was poverty and limited access to livelihood resources. For instance, because of limited income, participants discussed not having enough money to pay for transport to the facility to access health services. While some participants mentioned leaving early in the morning and walking for hours to travel to the facility, one mother said,

“*Last month when I stopped [coming to the clinic]*, *I did not come because of transport”*

[non-m2m participant #5].

Another frequently cited barrier was not having enough food to eat to counteract ART side effects. Most participants mentioned continuing to adhere to treatment despite such challenges, but one participant described how she often misses doses when she does not have enough food to eat:

“*We take the medication and at times you find that we have no food*. *We become weak*. *Our bodies are not strong”*

[non-m2m participant #31].

Economic disempowerment and financial dependence were often cited as barriers to care. Indeed, participants discussed a lack of financial support from partners and family members to overcome the indirect and opportunity costs related to engaging in PMTCT care. One participant reported that she was considered to be “*a waste of [her parent’s] money”* [non-m2m participant #2] after becoming pregnant and acquiring HIV, so her parents stopped supporting her and paying her school fees. The majority of m2m and non-m2m FGD participants reported that they dropped out of school when they became pregnant, or because they could no longer afford school fees due to competing economic priorities. The few participants reporting still being in school discussed challenges finding time to visit the health facility for care:

“*I fail to balance my time as to how I can go to school and at the same [time] go to collect the drugs”*

[m2m participant #29].

Stigma featured prominently as a recurring theme that touched upon many aspects of the adolescent mothers’ lives. One participant described how stigma impacted her ART adherence:

“*Sometimes people in the community discriminate against us*. *Sometimes we don’t take drugs because of what [hurtful things] they say”*

[m2m participant #29].

Participants also discussed self-stigma and feeling discouraged because they acquired HIV at such a young age. One expressed, “*I just wasted my life”* [non-m2m participant #3], and another said, “*I regret having contracted the virus; it doesn’t match with [my] age”* [m2m participant #30]. While several participants expressed a strong desire to return to school and find work to support themselves, fear of stigma and discrimination related to living with HIV, being a new mother, or both was often mentioned as a barrier to adolescent mothers returning to school. They described worrying that people will marginalize them because of their HIV status. One participant also reported that stigma adversely impacts access to work opportunities:

“*mostly when we ask for piecework they tell us that*, *‘Remember you have AIDS; you cannot manage to cultivate in my field’”*

[m2m participant #2].

### Facilitators of care and treatment for adolescent mothers living with HIV

Self-efficacy and personal motivation to stay healthy and keep their infants HIV-free was a highly cited facilitating factor for engaging and staying in care. One participant said, “*so when you take the medication you will see that your health is secured for a long time”* [non-m2m participant #26]. In terms of protecting her infant, one participant described her perseverance despite her fear of stigma, “*I threw [my medications] away because I knew that they would find me to have the disease [AIDS]…but I accepted the treatment*, *because I wanted to protect the unborn child”* [m2m participant #25].

Recurring themes of self-motivation, determination, and resilience were also apparent in frequent descriptions of participant efforts to attend clinic visits despite not having money to pay for travel expenses and collect their medications while at the clinic:

“*Even though the distance is long*, *when it’s time to collect our medication and there is no transport*, *we still walk and get there so long as we get the medication”*

[non-m2m participant #32].

Additionally, both m2m and non-m2m participants described taking their medications despite not having enough food to eat:

“*So food is scarce*. *But what can I do*? *I was told not to skip the medication*, *so I take it”*

[non-m2m participant #17].

Male partner support, when available from husbands and boyfriends, facilitated care engagement. Participants noted that their male partners often supported them in remembering to take their medication daily and encouraging them to stay in care. One participant stated, “*ever since I started*, *I have never skipped a dose because of the encouragement from my husband”* [m2m participant #23]. Another m2m participant discussed how her husband ensures that she takes her medication so that she and her infant remain healthy. While less commonly described, a few participants discussed how their husbands supported them by paying for their transport to the health facility and providing them with food. In some cases, participants mentioned parents and extended family members who supported them with treatment adherence and accompanied them to the facility to help them collect their medications and receive health services. However, there were instances in which male partners and family members provided no support. One participant explained how her husband left her after learning about her HIV status:

“*I disclosed to my husband and when I explained to him*, *he left me; he said that I should not concern him”*

[m2m participant #32].

Participants also acknowledged receiving support from health care workers, m2m mentor mothers, teen clubs, and support groups in the forms of encouragement, health education, adherence counseling, and psychosocial support. One non-m2m participant discussed how the health care workers counseled her that being HIV positive is not the end of her life and that she can continue living a healthy life if she adheres to ART:

“*The time I was I pregnant I went for the [HIV] test*, *and when I went there they told me the results*. *I was afraid and confused*, *and I pitied myself until I started crying right there*. *The health workers counseled me that it’s not the end of life*, *but the beginning of a new life”*

[non-m2m participant #6].

Another participant described challenges with health facility staff attitudes, but stated that the mentor mothers always encouraged her:

“*The mothers2mothers [mentor mothers] encourage you*, *but when you find a doctor who is not good hearted*, *it does not happen*. *You are left unattended*, *while those [mentor mother] women encourage you”*

[m2m participant #13].

She went on to further detail the encouragement she was receiving from mentor mothers:

“*if the mentor mothers were not giving us counseling*, *I could have been saying*, *‘today I will take medication but I will not take [them] tomorrow*.*’ But they encourage you to take the medication every day”*

[m2m participant #13].

### Preferences for PMTCT support services among adolescent mothers living with HIV

When asked what services HIV-infected adolescent mothers would like to receive to help them stay in care and keep their infants HIV free, participants frequently described wanting pragmatic support with food, transport, and livelihood assistance to meet their basic needs and facilitate ART adherence. Participants stated preferences for services and other sources of support focused on addressing underlying issues of poverty, food insecurity, and lack of income generating opportunities. One m2m participant [#30] stated, “*we are failing to get the basic needs because of lack of money”*. Numerous participants also expressed wanting assistance with traveling to the facility to collect their medication, and support to go back to school or start their own business to gain economic independence: “*I want business but I lack the capital to get started”* [m2m participant #22]. In addition to expressing a desire for greater access to material resources, all participants reported wanting home visits and household-level support for their PMTCT care. In terms of the type of household-level support, one participant said:

*“They should come home*, *at other times*, *as an individual*, *you can forget*, *or let me say that you think that*, *‘I am feeling fine this week*, *I will not take medication*,*’ then you discover the following week you start getting ill*. *So when they come*, *then they encourage you saying that you should be doing what*, *you should be taking medication every day*, *you should not miss any medication”*

[m2m participant #20].

A majority of participants cited the critical importance of healthcare providers safeguarding their privacy and confidentiality. Participants, who described collecting medication on Saturdays or in a separate queue or private area at the clinic, mentioned how those strategies allayed worry about being stigmatized. One participant detailed her desire for greater privacy as follows:

“*Like here at the clinic what I would like to change is the place where we sit waiting to get our medicine*. *Because we are able to see each other…when we meet with people from our village we get embarrassed*, *so if they could change the place so that we should not meet or face the other people [that would be good]”*

[non-m2m participant #6].

### Adapting the Mentor Mother Model to meet the needs of adolescent mothers living with HIV

When asked to reflect on ways to adapt the Mentor Mother Model to better meet their needs, most participants mentioned wanting to receive psychosocial support from someone with whom they could relate to personally and who would encourage them, particularly someone of a similar age, such as between the age of 18 and 24 years. In response to the idea of receiving support from a “peer” mentor mother, one participant said:

*“I would have loved to have a young [mentor mother] because we are at the same stage and also she has gone through [the same] situation*, *while [with an] older woman our ways of thinking would be different”*

[non-m2m participant #6].

Another participant described her desire for receiving psychosocial support from an adolescent mentor mother this way:

“*I would love if there was a young woman to encourage us*, *to ease our anxiety which we would have if we were alone”*

[m2m participant #7].

A few participants expressed concerns that a younger mentor mother may not know as much about the world or navigating PMTCT services as an older woman, or might not be able to keep their HIV status confidential:

*“I am able to discuss with these older people*. *I can be confident with these older people*, *but these young ones*, *they are fond of revealing secrets”*

[m2m participant #7].

Adolescent mothers repeatedly highlighted the importance of experience navigating the PMTCT cascade as a key qualification for any mentor mother. Participants noted that such experience is essential for ensuring that adolescent mothers relate to their mentor mothers. One participant framed the relevance of experience this way:

“*someone who has been taking the drugs for a long time [and] that knows the pain of taking the medication”*

[non-m2m participant #24].

Most participants commented that the ideal mentor mother would be a peer who:

“*[could] be helpful because she can counsel us based on her experience with HIV”*

[m2m participant #28].

Additionally, participants recommended that a “peer” mentor mother should visit them at their homes to encourage them and ensure that they are adhering to treatment:

“*When she comes she will be encouraging us not to feel tired about taking medication”*

[non-m2m participant #23].

## Discussion

Our qualitative findings illustrate the main barriers to engaging and retaining HIV-infected adolescent mothers and their infants in the PMTCT cascade in Malawi, and how existing PMTCT support programs can be tailored to better meet the unique needs of adolescent mothers living with HIV in sub-Saharan Africa. We provide some of the first qualitative descriptions from the region about the service delivery preferences of HIV-infected adolescent mothers, including their wish to receive support from trained lay providers in their peer group who have experience successfully navigating the PMTCT cascade and who may be younger than existing mentor mothers in traditional m2m programming.

Adolescent mothers living with HIV in Malawi encounter multiple barriers to care that include poverty, stigma, discrimination, gender inequality, and food insecurity. These barriers can be experienced by AGYW with or without m2m experience, and adversely affect mothers’ attempts to navigate the PMTCT cascade and ensure that they and their children stay healthy and HIV-free. To overcome these barriers, adolescent mothers living with HIV turn to facilitating factors such as their own personal motivation and resilience, as well as external support from male partners, family, health care workers, and mentor mothers in the form of information, counseling, encouragement, and material resources. However, such support is often insufficient to help them overcome the most entrenched structural and psychosocial barriers to HIV care, which are more frequently encountered by adolescent girls than their older HIV-infected counterparts [32–33]. While the availability of support programming, such as mothers2mothers, contributed to adolescent mothers feeling empowered to access PMTCT services, participants still expressed a desire for additional assistance in the form of more tailored and age-appropriate psychosocial support, material resources, and economic opportunities.

While previous research has revealed that HIV-infected adolescent mothers experience lower rates of ART initiation, retention along the PMTCT cascade, and HIV testing for their HIV-exposed infants, limited evidence describes barriers to these services in this population and how these barriers contribute to inferior maternal and child health outcomes [14–17, 34]. Our qualitative findings reinforce previous work from sub-Saharan Africa on health system barriers and identify the obstructive effects of care that is not patient-centered, involves harsh treatment by health workers, and does not protect the privacy and confidentiality of adolescent mothers living with HIV [32]. Additionally, and similar to other studies, we noted that psychosocial and structural barriers such as stigma, low socioeconomic status, and inadequate knowledge of PMTCT services and processes also pose a major barrier to adolescent PMTCT care engagement [35].

Our findings further highlight the need for evidence-based PMTCT programming that is tailored to the unique needs of adolescent mothers living with HIV and that offers support along the entire care cascade. While several studies have argued for additional research on adolescent-friendly PMTCT programming, few have evaluated existing programs from the perspective of adolescent beneficiaries [14–20]. Although not explicitly mentioned by our adolescent participants, issues of gender inequity underlined their reported experiences of discrimination, structural barriers to service access, and economic marginalization. Previous work from sub-Saharan Africa has documented the association between gender inequality for AGYW and their increased risk of intimate partner violence and adverse sexual and reproductive health outcomes, including incident HIV infection [36, 37]. The effectiveness of PMTCT programming for AGYW, particularly during the high-risk post-partum and breastfeeding periods [38], would benefit from comprehensive gender empowerment interventions [37, 39]. Deeply intertwined with gender inequality were participant descriptions of economic vulnerability and lacking sufficient financial support to meet their basic needs. This finding suggests a gap in research and programming around PMTCT-integrated socioeconomic interventions, such as cash transfer programs, micro-finance interventions, and income generating activities designed to economically empower HIV-infected adolescent mothers and their families.

Evidence from SSA on the association between financial incentives and other economic empowerment interventions and care retention for PMTCT clients is limited, especially for adolescent mothers living with HIV. Among adolescents living with HIV in South Africa, self-report of having enough money to travel to the clinic was one of five factors associated with increased retention in HIV care, along with sufficient clinic ART supply, accompaniment to the clinic, being treated well by healthcare staff, and taking time to talk to adolescents [40]. Available data from Tanzania and the Democratic Republic of Congo (DRC) suggest that conditional cash transfers and food assistance can support HIV-infected adult women to remain engaged in PMTCT care [41]. In DRC, women receiving cash transfers as part of a randomized controlled trial had higher retention in care and uptake of PMTCT-related services compared to women in the control group [42]. Additionally, a review of various social protection schemes highlights their potential to improve HIV outcomes among adolescents [43]. Preliminary results from a program in Jamaica suggest it may also be feasible to train life coaches to support adolescent girls to remain in PMTCT care and to access income generating activities [44]. While previous research suggests that financial incentives can improve PMTCT care retention and service uptake, results for other PMTCT outcomes have been less encouraging, with no effects seen on maternal ART adherence or virological suppression [45]. The effects of financial incentives on PMTCT and other HIV-related health outcomes may depend on a host of factors, including the population reached, incentive amount, whether it is conditional or unconditional, and the step targeted in the HIV prevention to care continuum [42, 46, 47]. Further research is needed to identify and assess interventions to pragmatically address the social determinants of poor HIV outcomes among adolescent mothers living with HIV, including financial incentives and social protection schemes to mitigate the poverty and economic inequality they face in SSA.

We identified several ways that existing PMTCT programming can be adapted to better meet the needs of adolescent mothers living with HIV. First, study participants expressed a preference for engaging with mentor mothers of a similar age with whom they could relate. Existing m2m programming can incorporate recruitment, training, and remuneration for a dedicated cadre of “adolescent peer” mentor mothers between the ages of 18 and 24 years who focus exclusively on engaging adolescent mothers as their clients. Second, based on participant responses, peer mentor mothers should have experience successfully navigating the PMTCT cascade, sustaining ART for at least 2 years, and surmounting those health-system, psychosocial, and structural barriers disproportionately affecting HIV-infected adolescent mothers—barriers such as dual stigma from being HIV-infected and a young mother, or the challenges of staying in school while simultaneously facing the new responsibilities of motherhood. Third, any adolescent mentor mother cadre must safeguard beneficiary privacy and confidentiality at all times, as a few participants described their concerns about inexperienced mentor mothers potentially not keeping sensitive health information secret. Promoting privacy confidentiality can be achieved through dedicated training and integrated supportive supervision and mentorship. Fourth, to facilitate confidentiality, promote care navigation, and provide psychosocial counseling, the same cadre of adolescent mentor mothers must engage beneficiaries with support both in the community as well as in the facility, serving as a bridge between home and clinic. Finally, to address barriers of poverty and gender inequality, adolescent mentor mothers can serve as a resource to link adolescent mothers to economic opportunities and interventions in their communities. Such interventions can be incorporated into existing programs, such as the mothers2mothers program, and funded by agencies focused on HIV, sexual and reproductive health, and social protection programming for AGYW. Funding support could come from the World Bank, United Nations agencies, or the PEPFAR *D*etermined, *R*esilient, *E*mpowered, *A*IDS-free, *M*entored and *S*afe (‘DREAMS’) partnership, as well as the Ministry of Health budget to support long-term sustainability.

While these recommendations are derived from the articulated needs, preferences, and experiences of our study participants in Malawi, the generalizability of our findings, and other elements of our study, have limitations. First, because we recruited participants at health facilities, we may not have been able to reach AGYW without access to, or who completely disengaged from, public health services. Second, at two of the sites, we were only able to enroll three participants per FGD, which can limit the diversity of opinions and input available for those FGD data. Third, while we did successfully enroll adolescent mothers as young as 15 years (n = 4 participants), the perspectives of the youngest adolescent mothers may have been underrepresented. Finally, as this implementation research was intended to help adapt the m2m Mentor Mother Model, our results may not be fully generalizable to all PMTCT support programs. However, we expect our findings to have general relevance to PMTCT programs involving other community-based or task-shifted lay health providers, such as peer educators and community health workers.

## Conclusions

In conclusion, adolescent mothers living with HIV in SSA face multi-level barriers to PMTCT care and express preferences for peer-driven support services. The desired support services should be delivered by young women who are mothers living with HIV themselves, relatable, and uphold privacy and confidentiality. These women should offer services that are non-judgmental, bridge communities and facilities, and address barriers of stigma, poverty, health system complexity, and food insecurity, among others. Future mentor mother programs focused on adolescent mothers living with HIV should incorporate socioeconomic interventions, such as cash transfers, income generating activities, or other social protection approaches that pragmatically lower structural barriers to HIV care. To engage and retain adolescent mothers in the PMTCT cascade, they need opportunities to build their own livelihoods and meet their basic needs, feel empowered to stay in care to promote their own health and that of their children, and access to high-quality, longitudinal, peer-led psychosocial support touching on multiple aspects of their lives.

## Supporting information

S1 File Semi-structured focus group discussion guides in English and Chichewa, Yao, and Tumbuka. (PDF)
