# Supplementary material for: “I would love if there was a young woman to encourage us, to ease our anxiety which we would have if we were alone”: Adapting the Mothers2Mothers Mentor Mother Model for adolescent mothers living with HIV in Malawi
Source: PLoS One. 2019 Jun 7;14(6):e0217693. doi: 10.1371/journal.pone.0217693 (PMC6555548; doi:10.1371/journal.pone.0217693)
Supplement: S1 File — (PDF) [file pone.0217693.s001.pdf]

Annex 1: Adolescent Mother Needs, Attitudes, and Preferences
Focus Group Discussion Guide

Introductory Script (follows written informed consent process):

Please read: ["Hello. Thank you for agreeing to participate in a focus group discussion today. My name is _________________________ and I will be facilitating today's discussion. My friend/colleague here is ________________________ and he/she will be taking notes.

Your participation in today's focus group will help us gain valuable insight into ways to help young mothers and their babies to keep going to the clinic, taking their ARVs/medicines, and staying healthy.

This will be a group discussion. It's not an interview where we ask a question and each person answers the question and we move on to the next one. Instead, we'll be putting topics on the table and the idea is for everyone to participate in the discussion with each other. You have all been assigned a number – please mention this each time you speak.

We are interested to hear about the group's thoughts, feelings, and experiences regarding services in the clinic and the community to stop HIV from being passed from mothers to children. These types of services are sometimes called 'PMTCT' services. We also want to hear everyone's opinions, preferences and recommendations for how best to support young mothers living with HIV from the time they become pregnant until when they are done breastfeeding their babies. We want to hear both similarities and differences within the group. There are no right or wrong answers to these questions. You do not have to answer questions if you do not want to. Please free to ask questions if something is unclear.

I want to remind you that the information shared during the discussion today is confidential. That is, we will not share anything said during the discussion today with anyone outside this space/ room, and what you say will not be connected back to you. While the information gathered during this discussion will be combined with information from other discussions and shared with the Ministry of Health and other organizations to make PMTCT services better, no-one will know who said what, when things were said or where things were said.

Do you have any questions before we begin?"]

[NB: informed consent forms should already be signed and collected]

General questions about PMTCT from the client/ patient perspective

Part 1 - Background information (20 minutes):

a) Before we talk about your thoughts, we will go around the circle and have everyone introduce themselves. Please tell us your age, where you are from, and how many children you have.
b) Could you tell us how long you have been coming to this/ the clinic in your community?
c) What do you know about mothers2mothers?

*[**PURPOSE OF QUESTIONS:** Some icebreakers to establish rapport; a non-threatening introduction]*

d) "Based on your experience being pregnant/ having a baby, what do you think are the things that affect the health of young mothers living with HIV and their babies in a good way?

[PROBES]

- Age/ gender
- Partner/ husband / family
- Social situation (e.g. work, education)
- Food/ nutrition
- Travel/ transport
- Healthcare quality/ availability
- Support (both at the clinic and in the community)
- Other

e) Based on your experience being pregnant/ having a baby, what do you think are the things that affect the health of young mothers living with HIV and their babies in a bad way"?

[PROBES]

- Age/ gender
- Stigma (both internal and external)
- Partner/ husband / family
- Social situation (e.g. poverty, joblessness, education)
- Food/ nutrition
- Travel/ transport
- Healthcare quality/ availability
- Support (both at the clinic and in the community)

[PURPOSE OF QUESTION:** To determine client perceptions and experiences of what factors impact their health and access to health care. NB: These could be POSITIVE or NEGATIVE factors. We are interested in both.**]

f) How do these factors affect the health of young mothers living with HIV and their babies?

***[PURPOSE OF QUESTION:** To understand HOW the issues described above are perceived to affect health.]*

Part 2 - Previous Experiences with Health Care and PMTCT Services (30 minutes):

Please read: ["We are now going to talk about healthcare and PMTCT services in your community…"]

a) "How do you go about getting health services for you and your baby when you need them?"

***[PURPOSE OF QUESTION:** To establish factual detail about the processes adolescent mothers actually go through when accessing healthcare in their community.]*

b) "Can you tell us about your experiences getting tested for ***HIV at the clinic/ during antenatal***?"

[PROBES]

- Where is the best place to get HIV testing and counseling?
- Who do you trust to do the HIV testing and counseling?
- What helps young mothers get HIV testing and counseling?

***[PURPOSE OF QUESTIONS:** To document adolescent mothers' experiences with HIV testing and understand adolescent mothers' perceptions of enabling factors for HIV testing.]*

c) "What challenges, if any, have you faced in starting ***ARVs*** for the first time? In taking ARVs after starting? Why?"
*[**Note for RAs**: Discuss with participants the process of being offered and starting ARVs for the first time, how they felt, and the challenges they faced at that point and since then with continuing to take ARVs.*
[PROBES]:
  - Stigma
  - Attitudes
  - Transport
  - Poverty
  - Time/ Competing responsibilities
  - Partner/ Family
  - Support structures/ enablers
  - Other issues

*[**PURPOSE OF QUESTIONS:** To understand how adolescent mothers access ARVs and document their experience of barriers and enablers to regularly accessing ARVs]*

d) "What do you feel are the major challenges you have faced in getting your baby tested and treated for HIV? Why?"
  - Experience of going for infant testing and getting infant test results back
  - Support structures and enablers for infant testing
  - Access to ARVs for infants/ young children

e) What are the things that you would like to improve about the ***health services*** you or your baby receive?
[PROBE]
  - Availability/ Access
  - Health worker attitudes
  - Support systems

*[**PURPOSE OF QUESTION:** To establish those factors, from the perspective of adolescent mothers, that are most important for improving access or quality of care]*

Part 3 - Experiences with m2m mentor mothers (35 minutes):
[SKIP if FGD is being conducted with adolescent mothers who do not currently or have not previously received m2m services.]

Please read verbatim: ["I would now like to ask some questions about your experiences with mentor mothers."]

a) "Can you please describe in your own words who are mentor mothers and what you think they do at the clinic and in your community?"

*[**PURPOSE OF QUESTION:** To determine adolescent mothers' understanding of who mentor mothers are and what they do, including key activities]*

b) "From your own experiences, has m2m/ mentor mothers changed the way you personally get healthcare for you or your baby? Please describe any positive or negative changes."
[PROBE]
  - Access to health information
  - Antenatal care

- HIV testing and counseling
- Partner involvement/ couples counseling and testing
- ART initiation/ Option B+
- Adherence counseling/ support for taking ARVs
- Attended delivery
- EID/ infant DNA PCR testing
- Breastfeeding counseling
- Under 5/ immunization/ nutritional status monitoring

***[PURPOSE OF QUESTION:** To establish specific detail on which aspects of PMTCT service delivery has changed positively or negatively because of m2m or a mentor mother]*

> ***Note to RAs**: It is possible (although hopefully unlikely) that participants have not experienced or perceived any changes. If this is the case, and they re-iterate that they don't think there were changes, do not keep probing as it is disrespectful. However, if they mention such changes in the context of another question – it is OK to probe at <u>that</u> point, as the participant has obviously 'made sense' of this idea in relation to a different question.]*

c) How is the care and support you have received from mentor mothers different from what you receive from healthcare workers?

***[PURPOSE OF QUESTION:** To establish specific examples of where adolescent mothers have been unable to access or take their ARVs and the factors contributing to these cases]*

<u>Part 4 – Preferences for Services (35 minutes):</u>

a) "What sorts of help or services would you like to receive to help with staying healthy during pregnancy and while breastfeeding, and keeping your baby free of HIV?" Why or why not? ***[PROBES]***
   - Counseling / social work services
   - Support group or one-on-one meetings
   - Help / support / reminders to take ARVs daily
   - Talking to male partner or family
   - Airtime
   - Transportation to and from the clinic
   - Food/ nutritional supplementation
   - Other

***[PURPOSE OF QUESTION:** To establish examples of PMTCT services adolescent mothers may want and why they may be wanted]*

b) "Would you consider having another mother like yourself who is young and also living with HIV work with you to make sure you receive all the services you need to stay healthy and keep your baby HIV free? Please explain why or why not."

c) "If you would consider having another young mother living with HIV support you during pregnancy and after delivery, what sorts of services would you like such a person to offer?" ***[PROBES]***
   - Adherence counseling
   - Couples counseling and testing
   - Refills of ARVs and other medications
   - Calling/ SMS you with reminders about appointments

- Visit you at home periodically to make sure you and your baby are healthy
- Provide a food package/ nutritional assistance
- Provide information about:
    - Positive living
    - Adherence
- Provide counseling for:
    - How to talk to partners and family about HIV, your pregnancy, etc.
    - Mental health problems / depression
    - Reducing alcohol / substance use
- Provide information on educational / training opportunities in the community
- Other

***[PURPOSE OF QUESTION:** To establish specific examples of PMTCT support services adolescent mothers may want]*

d) "What sorts of feature would make it more likely for you to interact with another HIV-positive mother in your community and each time you visit the clinic?"
[PROBES]

- Age
    - Adolescent (19 years of age or younger)
    - Young adult (ages 20 through 24)
    - Adult (ages 25 to 50)
    - Senior (over the age of 50)
- Mentor experience
    - Trained as a peer health counselor
    - Successfully accessed PMTCT services and kept herself and her baby healthy
    - Length of time living with HIV
- Encounter/ Meeting location
    - Virtual
        - Facebook/ Internet
        - Phone/ SMS/ WhatsApp
    - Venue-based
        - Clinic/ health facility
        - Home
        - School
        - Place of worship
        - Other
- Encounter schedule / hours
- Encounter duration
- Other

***[PURPOSE OF QUESTION:** To establish specific attributes of mentor mothers and their encounters with mentor mothers that might be preferable to adolescent mothers]*

e) "Is there anything else you would like to share before we finish?"
[PROBE]

- Unmentioned issues

***[PURPOSE OF QUESTION:** To provide participants with the opportunity to share any other information that may be relevant to how they access or would like to be supported for PMTCT services either now or in the future]*

Adolescent Mother Needs, Attitudes, and Preferences
Focus Group Discussion Guide

Introductory Script (follows written informed consent process):

Chonde werengani mokweza: ["Muli bwanji. Zikomo kwambiri povomereza kutenga nawo mbali mu zokambirana za pagulu lero. Dzina langa ndi ________________________ndipo ndikhala ndi mzanga________________________ndipo iye azilemba zonse zomwe tikhale tikukambirana.
Kupezeka kwanu pa zokambirana za lero kutithandiza kupeza nzeru zozindikira njira zothandizira amayi achitsikana ndi ana awo kuti apitilize kupita kuchipatala, kumwa ma ARV, ndi kukhalabe ndi moyo wa thanzi.

Zokambirana izi zikhala za pagulu. Sizokambirana zomwe tikhala tikufusunsa mafunso okhaokha ayi. M'malo mwake, tikhala tikupereka ganizo lomwe wina aliyense akuyembekezereka kuti aikepo Maganizo ake pamene tikukambirana wina ndi mzache. Aliyense wa inu wapatsidwa nambala choonde tchulani nambala yanu nthawi iri yonse yomwe mwapatsidwa mpata kuti muyankhule.

Tikufuna timve maganizo anu mong gulu kapena zomwe mwakomana nazo zokhudza zithandizo zoperekedwa mu chipatala ndi m'dela zothandiza kuthetsa kupatsira kachirombo kuchokera kwa amayi kupita kwa ana. Zithandizozi zimadziwika ndi dzina la mchingerezi la 'PMTCT'. Tikufunanso timve maganizo anu monga gulu, kukonda kwanu komanso malangizo anu pa momwe tingathandizire moyenera amayi achichepere amene ali ndi kachirombo ka HIV kuchokera pa nthawi imene atenga mimba kufikira pamene asiya kuyamwitsa ana awo. Tikufuna tidziwe kufanana kapena kusiyana muzinthu izi pa gulu lino. Kukumbukirani kuti palibe yankho loona komanso lolondola mu zokambirana zathuzi. Muli ndi ufulu kusayankha mafunso ngati mufuna kutero.

Mongokumbutsana, uthenga umene titagawane pano ndi wa chinsisi. Choncho, zokambirana zathu za lero sizidzanenedwa kwa aliyense amene asali mchipinda chino komanso zomwe mungatifotokozere sizidzadziwika kuti zayankhulidwa ndi inu. Uthenga umene utatengedwe mu zokambiranazi udzaphatikizidwa ndi uthenga wina ochokera ku zokambirana zina ndi kuperekedwa ku unduna wa za umoyo ndi mabungwe ena pofuna kupititsa patsogolo ndondomeko zokhudza kupewa kupatsira kachirombo kuchokera kwa amayi kupita kwa mwana ndipo palibe amene azadziwe za anthu, nthawi ndi malo amene zokambiranazi zimachitikira..

Muli ndi funso lililonse tisanayambe zokambiranazi?"]

<u>Mafunso okhudza maganizo a otenga nawo mbali pa ndondomeka yoteteza mwana kuti asatengere kachirombo kuchokera kwa mayi</u>

<u>Gawo loyamba – Zokhudza otenga nawo mbali (20 minutes)</u>

a) Tisanayambe kukambirana za Maganizo anu, wina aliyense afotokoza kuti iye ndani. Chhonde tiuzeni dzina lanu, zaka zanu, kumene mwacjokera, ndipo nambala ya ana omwe muli nawo.
b) Kodi mungatifotokozere kuti ndi ntahwi yaitali bwanji yomwe mwakhala mukubwera ku chipatala chino / chipatala cha m'muzdi mwanu?
c) Kodi mukudziwa zotani zokhuza mother2mothers

d) Malingana ndi zomwe wakomana nazo m'mene munali oyembekezera kapena kukhala ndi mwana oyamba, kodi mukuganiza ndi zinthu ziti zomwe zimakhudza thanzi la amayi achichepele omwe ali ndi kachilombo ka HIV ndi ana awo munjira yabwino?

[Zoti Mufunse]

- Zaka/mwamuna kapena mkazi
- Mwamuna wokhal limodzi ngati banja / chibwenzi
- Zinthu zotikhudza m'moyo wathu (monga., ntchito, maphunziro)
- Chakudya
- Mayendedwe
- Ubwino ndi kupupezeka kwa chithandizo cha zaumoyo
- Ndomdomeko zosiyana siyana zothandiziraZina

e) Malingana ndi zomwe wakomana nazo m'mene munali oyembekezera kapena kukhala ndi mwana oyamba, kodi mukuganiza ndi zinthu ziti zomwe zimakhudza thanzi la amayi achichepele omwe ali ndi kachilombo ka HIV ndi ana awo munjira yolakwika?

[Zoti Mufunse]

- Zaka/mwamuna kapena mkazi
- Kusalidwa
- Mwamuna wokhal limodzi ngati banja / chibwenzi
- Zinthu zotikhudza m'moyo wathu (monga., umphawi, ntchito, maphunziro)
- Chakudya
- Mayendedwe
- Ubwino ndi kupupezeka kwa chithandizo cha zaumoyo
- Ndomdomeko zosiyana siyana zothandizira
- Zina

[PURPOSE OF QUESTION:** To determine client perceptions and experiences of what factors impact their health and access to health care. NB: These could be POSITIVE or NEGATIVE factors. We are interested in both.**]

f) Kodi mukuganiza kuti zinthuzi zimakhudza azimayi achichepele amene ali kachilombo ka HIV ndi ana awo munjila yotani?

***[PURPOSE OF QUESTION:** To understand HOW the issues described above are perceived to affect health]*

Gawo lachiwiri – Zimene mukudziwa zokhudzana ndi thandizo la zaumoyo ndi kuteteza mwana kuti asatengere kachilombo ka HIV (30 minutes)

Chonde werengani mokweza: ["Tsopano tikambirana zokhudzana ndi thandizo la zaumoyo ndi zina zothandiza mwana kuti asatengere kachilombo ka HIV kuchokera kwa mayi m'dela lanu…"]

a) "Kodi zimakhala bwanji pamene inu kapena mwana wanu mwafuna thandizo la zaumoyo, ndondomeko yake imayenda bwanji kuti mupeze thandizolo?"

*[**PURPOSE OF QUESTION:** To establish factual detail about the processes adolescent mothers actually go through when accessing healthcare in their community.]*

b) Mungatiuzeko m'mene zinalili pamene mumayezetsa kachilombo ka HIV kuchipatala kapena pamene mumakayamba sikelo

[PROBES]

- Kodi malo abwino kuyezetsera ndi kuti?
- Kodi inu mumamukhulupilira ndani kuti akuyezeni?
- Kodi chimene chimathandizira ndi chiyani kuti amayi achichepere ayezetse magazi komanso kulandira uphungu?

*[**PURPOSE OF QUESTIONS:** To document adolescent mothers' experiences with HIV testing is done and understand adolescent mothers' perceptions of enabling factors for HIV testing]*

c) "Mungatiuzeko zimene mwakumana nazo pamene mumayamba kulandira mankhwala ama ARV kwa nthawi yoyamba? Kupitiliza kumwa ma ARV? Chifukwa chiyani? "

*[**Note for RAs**: Discuss with participants the process of being offered and starting ARVs or the first time, how they felt, and the challenges they faced at that point and since then with continuing to take ARVs.*

[PROBE]

- Zaka/mwamuna kapena mkazi
- Mwamuna wokhal limodzi ngati banja / chibwenzi
- Zinthu zotikhudza m'moyo wathu (monga., ntchito, maphunziro)
- Chakudya
- Mayendedwe
- Ubwino ndi kupupezeka kwa chithandizo cha zaumoyo
- Ndomdomeko zosiyana siyana zothandizira

Zina

*[**PURPOSE OF QUESTIONS:** To understand how adolescent mothers access ARVs and document their experience of barriers and enablers to regularly accessing ARVs]*

d) Kodi mukuganiza kuti ndi zovuta zotani zomwe mwapezana nazo pomuyezetsa mwana wanu kachilombo ka HIV ndikulandira chithandizo? Ndichifukwa chiyani?

- Zomwe mwapezana nazo pomuyezetsa mwana ndikulandira zotsatira
- Zinthu zothandizira kuyeza ana magazi
- Kupezeka kwa ma ARV a wana

e) Kodi, ndi zinthu ziti zimene mukufuna zitasintha zokhudza zithandizo zaumoyo zimene inu ndi mwana wanu mumalandira?

[Zoti mufunse]

- Kupezeka kwa zithndizozi
- Makhalidwe a anthu ogiwra ntchito zachipatala
- Zinthu zina zothandizira

*[**PURPOSE OF QUESTION:** To establish those factors, from the perspective of adolescent mothers, that are most important for improving access or quality of care]*

Gawo lachitatu – Zimene mukudziwa zokhudza amayi othandizira a mayi anzawo (mother to mother) (35 minutes) ***[SKIP if FGD is being conducted with adolescent mothers who do not currently or have not previously received m2m services.]***

Chonde werengani mokweza: ["Tsopano ndikufuna ndifunse mafunso okhudza zimene mukudziwa za amayi othandizira."]

a) "Kodi mugandifotokozere kuti amayi othandizirawa ndi ndani ndipo mukuganiza kuti amapanga chani pachipatala ndi m'dela lanu?"

*[**PURPOSE OF QUESTION:** To determine adolescent mothers' understanding of who mentor mothers are and what they do, including key activities]*

b) Molingana ndi zomwe mwakomana nazo, kodi kupezeke kwa m2m/amayi othandizirawa kwasintha mmene inu mumapezera chithandizo cha zaumoyo kwa inu ndi mwana wanu? Choonde fotokozani zabwino kapena zofooka.

Zoti mufunse]

- Kapezedwe ka unthenga wa zaumoyo
- Chisamaliro chakusikelo
- Kuyesedwa kachilombo ka HIV ndi uphungu
- Kutenga nawo mbali/Kuyesedwa ndi kulandira uphungu pamodzi ndi okondedwa wako
- Kuyamba kulandila mankhwala a maARV
- Kulandira uphungu ndi chithandizo chikhudzana ndi ma ARV
- Kuthandizidwa pobereka
- Kuyesedwa magazi kwa ana oyamwa amene amayi awo ali ndi kachilombo ka HIV
- Uphungu wa kayamwitsidwe ka mwana
- Kuona za makulidwe ndi thanzi la ana osapitilila zaka zisanu

*[**PURPOSE OF QUESTION:** To establish specific detail on which aspects of PMTCT service delivery has been changed positively or negatively because of m2m or a mentor mother]*

*<u>**Note to RAs**</u>: It is possible (although hopefully unlikely) that participants have not experienced or perceived any changes. If this is the case, and they re-iterate that they don't think there were changes, do not keep probing as it is disrespectful. However, if they mention such changes in the context of another question – it is OK to probe at <u>that</u> point, as the participant has obviously 'made sense' of this idea in relation to a different question.]*

c) Kodi pali kusiyana kotani pakati pa chithandizo chomwe mwalandira kuchokera ku mother 2mother kuyerekeza ndi chomwe mwalandira kuchokera kwa ogwira ntchito a za umoyo?

*[**PURPOSE OF QUESTION:** To establish specific examples of where adolescent mothers have been unable to access or take their ARVs and the factors contributing to these cases]*

Gawo lachinayi – Chithandizo chimene mungafune (35 minutes)

a) "Kodi ndi chithandizo chotani chimene mungakonde mutalandira chokuthandizani kukhala moyo wabwino pamene muli oyembekezera ndi poyamwitsa ndi kuthandiza mwana wanu kuti asatengere kachilombo ka HIV?" Chifukwa chiyani?

[PROBES]

- Chithandizo cha ntchito zosiyanasiyana za achipatala, mavoluntiya.
- Kukumana kwa magulu othandizana kapena zokambirana pakati pa antu awiri
- Kuyankhulana ndi mwamuna kapena banja
- Mayunitsi
- Mayendedwe opita ndi kuchokera kuchipatala
- Zakudya zowonjezera
- Zina

***[PURPOSE OF QUESTION:** To establish examples of PMTCT services adolescent mothers may want and why they may be wanted]*

***[PURPOSE OF QUESTION:** To understand HOW and WHY the services mentioned may be of help under the unique circumstances faced by HIV-positive adolescent mothers]*

b) Kodi mungafune patapezeka mzimayi wina wachichepere ngati uniyo amene alinso ndi kachilombo ka HIV kuti muzithandizana naye poonetsetsa kuti mukulandira chithandizo chonse chokuthandizani kukhala a thanzi ndi kuteteza mwana wanu kuti asatengere kachilombo ka HIV? Chonde fotokozani ngati ndi choncho kapena ayi." ***[If NO, SKIP to 4e]***

c) Kodi mutati mwasankha kukhala ndi mayi wina wachichepere yemwe ali ndi kachilombo ka HIV woti azilimbikitsana nanu pa nthawi imene muli ndi pakati komanso mutabeleka, ndi chithandizo chanji chimene mungafune kuti mayi nzanuyu azikupatsani

[PROBES]

- Uphungu wa kamwedwe ka makhwala koyenelera
- Uphungu ndi kuyezetsa magazi kwa banja
- Katengedwe ka ma ARV ndi mankhwala ena
- Kukuyimbilani kapena kukutumizilani uthenga pa lamya okukumbutsani za masiku amene mukuyenela kukapezeka kuchipala
- Kukuyenderani kunyumba kwanu mwa nthawi ndi nthawi kuti tiwone ngati inu ndi mwana wanu mulibwino
- Kukupatsani kaphukusi ka zakudya kapena chithandizo cha kadyedwe kopatsa thanzi
- Kukupatsani uphungu wa:
  - Kukhala moyo wachiyembekezo
  - Kutsata uphungu wakuchipatala
- Kupeleka uphungu wa izi:
  - M'mene tingayankhulanire ndi achikondi komanso banja zokhudza HIV ndi pakati pomwe munali/muli napo.
  - Kusokonezeka k wa m'malingaliro/ kupsinjika
  - Kuchepetsa kamwedwe ka mowa/kugwilitsa ntchito mwankhala ozunguza bongo
  - Kukupatsani uthenga wa maphunziro/kapena mwai wophunzira umene unga pezeke m'dela lanu
  - Zina

***[PURPOSE OF QUESTION:** To establish specific examples of PMTCT support services adolescent mothers may want]*

d) "Kodi ndi zinthu ziti zimene zingapange kuti inu mukhale wothekera koposa kuti inu mugwire ntchito bwino ndi mayi nzanu amene ali ndi kachilombo ka HIV m'mudzi mwanu kapena nthawi ina iriyonse yomwe mwabwera kuchipatala?"

Zoti mufunse]

- Zaka za munthu
    - Wachisodzela (zaka 19 kapena kuchepelapo)
    - Wachinyata (zaka-20 mpaka 24)
    - Akulu(zaka 25 – 50)
    - Wachikulire (opyola zaka 50)
- Kudziwa bwino kwa mai wokuthandizirani
    - Akhale wophunzitsidwa
    - Anakhalapo mundondomeko ya PMTCT ndipo iye ndi mwana wake ali ndi moyo wa thanzi
    - Kutalika kwa nthawi imene munthuyo wa khala ndi kachilombo ka HIV
- Malo amene mungakomane
    - Kukomana mosaonana
        - Makina a internet kapena facebook
        - Lamya/uthenga/whatsApp
    - Kukomana pa malo
        - Kuchipatala
        - Kunyumba
          Ku sukulu
        - Ku malo opempherera
        - Malo ena
- Nthawi yokumanirana
- Kutalika kwa nthawi yakukomana
- Zina

***[PURPOSE OF QUESTION:** To establish specific attributes of mentor mothers and their encounters with mentor mothers that might be preferable to adolescent mothers]*

e) "Pali chilichonse chimene mukufuna munene tisanamalize?"

[PROBE]

- Monga zina zimene sizinakambidwe zokhudza umoyo

***[PURPOSE OF QUESTION:** To provide participants with the opportunity to share any other information that may be relevant to how they access or would like to be supported for PMTCT services either now or in the future]*

Adolescent Mother Needs, Attitudes, and Preferences
Focus Group Discussion Guide

Introductory Script (follows written informed consent process):

Chonde werengani: [“Muli wuli. Yewo chomene pakuzomela kutolapo lwande pa vyakudumbilana pagulu mwanyahuno. Zina lane ndine _______________________. Ndine nidangirilenge vyakudumbilana vinthu vya mwahuno. Munyane uyu ni __________________ ndipo walembenge ivyo tidumbiranenge pano.

Kutolapo lwande kwinu pa vyakudumbirana vinthu mwahuno vitovwirenge kusanga nthowa zakovwirila bamama wadokowadoko na bana bawo kulutilizganga kuluta kuchipatala, kumwanga ma ARVs nakunkhalanga wanthazi.

Ivi viwenge vyakudumbirana paguru. Timufumbaninge yayi waliyose payenkha payenkha fumbo kuti muzyole, kweni tiyowoyenge mitu ya vyakudumbilana, ndipo waliyose watolepo lwande . mose mwapika nambala, iyo muyizunulenge pala mukhumba kuyowoya.

Tikhumba kupulika maghanoghano yinu nge gulu, panji ivyo mwajumphamo kunkhwasya wovwiri wakupelekeka ku chipatala na muvikaya vakuvwila kumazga kupilana kachibungu kufuma kwa bamama kuluta kwa bana. Ivyo pachizungu vukumanyika kuti ‘PMTCT’. Tikukhumbaso kupulika maghanoghano yinu mose, kutemwa kwinu kweniso uphungu winu pa umo tingavwirila makola bamama wachichipele awo wali nakachibungu ka HIV kufumila panyengo iyo watolela nthumbo mpaka kufikila panyengo iyo walekela kuwonkhisya bana bawo. Tikukhumba timanye vinthu ivyo vikuyana kweniso kupambana pa gulu pano. Palije mazgolo awo ngaunenesko panyake ghaboza.i. Muna wanangwa kuleka kuzgola mafumbo pala mwakhumba kuchita ntheula. Muli na ufulu kufumba fumbo pala mundapulikiske.

Mwakukumbuskana, uthenga uwu tigawanenge pano ngwachisisi. Mwantheula vyakudumbilana vithu timuphalilenge waliyose yayi uyo walimuno ivyo muyowoyenge pano vizamumanyikwa chala kuti mkayowoya ndimwe. uthenga uwo titolenge pano muvyakudumbilana vyithu vizamusazgana na vyamagulu yanyake nakupeleka ku unduna wa za umoyo na mabungwe yanyakhe pakukhumba kulutiska panthazi ndondomeko zakukhwaskana zakupewela kupilana kachibungu kufuma kwa bamama kuluta kwa bana ndipo palije uyo wazamumanya vya wanthu nyengo na malo awo vyakudumbirana vikuchitikila.

[“Pambere tindayambe pali fumbo lililonse?”]

<u>Mafumbo wakukhwaska maghanoghano gha wakutolapo lwande pandondomeko yakuvikilila bana kutolela kachibunbu kufumila kwa bamama</u>

<u>Gawo lakwamba – vyakukwaska wakutolapo nawo lwande(20 minutes)</u>

a) Pambele tindayambe kuyowoya vyamayanoyanoyinu, waliyose wayowoye zina lake, vyaka, uko mukufumila, nawana awo mulinawo.

b) Mungatimpharira utali wanyengo uwo mwankhala mukwiza kuchipatala chino?
c)

*[**PURPOSE OF QUESTIONS:** Some icebreakers to establish rapport; a non-threatening introduction]*

d) Nawukwantha winu wakuwapo na nthumbo kweniso na mwana, , vinthu vini viwemi ivyo mukuyanayana kuti vikukhwaska wamama wadokowadoko awo wali nakachibungu ka HIV nawana bawo ?

[PROBES]

- Vyaka/mwanakazi panji mwanalume
- Mfumu winu/wachibale
- Vyakukhumbika pa moyo wamunthu nge ntchito, sukulu
- Vyakulya
- Mayendelo
- Kusangika kwa chisamarilo cha za umoyo
- Wovwiri wakunyumba na wakuchipatala
- Vinyake

e) Pakulingana na unkhwantha winu wakuwapo na nthumbo/mwana, kasi mukughanayana kuti nivinthu mbuni ivyo vikunkhwaska bamama wadokowadoko awo walina HIV kweniso bana bawo munthowa iheni?

[PROBES]

- Msinkhu/mwanakazi panji mwanalime
- Kusankhana (pa banja pinu nakuwalo)
- Mfumu winu/wachibale
- ukavu
- Kuwa muchoko
- Kuwa mwanakazi
- Wakutemweka wini/ mfumu winu
- Ukavu
- Kukhala pantchito olo yayi
- Masambilo
-
- vyakulya
- vyakayendelo
- Kusangika kwa chisamalilo chiwemi cha umoyo
-
- Wovwiri wakuchipatala na wamuvikaya

*[**PURPOSE OF QUESTION:** To determine client' perceptions and experiences of what factors impact their health and access to health care. NB: These could be POSITIVE or NEGATIVE factors. We are interested in both.]*

f) kasi vinthu ivi viku khwaskaso umoyo wa bamama wadokowadoko awo wali na HIV na bana bawo ?

-

*[**PURPOSE OF QUESTION:** To understand HOW the issues described above are perceived to affect health]*

Gawo lachiwiri – ivyo mukumanya kukhwaska wovyili wazaumoyo na kuvikilila bana kuti wangatolelanga kachibungu ka HIV (30 minutes)

Chonde werengani : ["sono tidumbilanenge vyakukwaska wovyili wazaumoyo na kuvikililo ka bana kuti wangatolelanga kachibungu ka HIV kufumila kuchikaya chinu…"]

a) "kasi vikuwa wuli pala imwe panyake mwana winu mukukhumba wovwiri wa zaumoyo, ?"

*[**PURPOSE OF QUESTION:** To establish factual detail about the processes adolescent mothers actually go through when accessing healthcare in their community.]*

b) "Mungatimphalilako na umo vikawira apo mukayezeska kachibungu ka HIV kuchipatala panyake uko mukayambanga sikelo"

[PROBES]

- Kasi ninkhuni kuwemi uko mungayezgeska HIV nakusanga umphungu
- Kasi mukugomezga njani kuti wamuyegzeni HIV naku pani uphungu?
-
-
- vikuwovwirani nivichi bamama wadokowadoko kuti wayeziske HIV nakupokela uphungu?
-

*[**PURPOSE OF QUESTIONS:** To document adolescent mothers' experiences with how HIV testing and understand adolescent mothers' perceptions of enabling factors for testing.]*

c)"masuzgho wuli (pala walipo) awo mukasangana nawo pakwamba kumwa ma ARVs kwakwamba? Pukumwa ma ARVs pambere kuti mwambapo kale kumwa? Chifukwa?"

*[**Note for RAs**: Discuss with participants the process of being offered and starting ARVs For the first time, how they felt and the challenges they faced at that point and since then with continuing to take ARVs.*

[PROBE]

- Kusakhika
- Makhalilo
- kayendelo
- Ukavu
- Nyengo/mawudindo wakupokana pokana
- Wakutemweka winu
-

*[**PURPOSE OF QUESTIONS:** To understand how adolescent mothers access ARVs and document their experience of barriers and enablers to regularly accessing ARVs]*

d) "Mukughanaghana kuti masuzgo yakuluyakulu ni mbani awo mulikukumana nawo pakunkhumba kuyezgeska nakupokela uwovwiri wa HIV? Chifukwa?

- Unkhwantha wakuluta kukayezgeska HIV mwana winu nakutola vyakulondezga vyake

- Malo wakuyezgeskela wana HIV
- Kusangika kwa ma ARV wa bana

e) nivinthu wuli ivyo mungatemwa kuti visinthike vyakunkhwaska uwovwiri wazawumoyo wa imwe na mwana winu?

[PROBES]

- kusangika/kuwasanga
- Nkhalo yawakugwira ntchito kuchipatala
- Chiwovwiri

*[PURPOSE OF QUESTION: **To establish those factors, from the perspective of adolescent mothers, that are most important for improving access or quality of care]***

Gawo lachitatu – ivyo mukumanya vyakukhwaska bamama awo wakovwila ku bungwe la m2m (35 minutes)

[SKIP if FGD is being conducted with adolescent mothers who do not currently or have not previously received m2m services.]

Chonde werengani mwakukwezga: ["nimufumbaninge naumo mukumanyila vyakukwaska bamama wakovwira"]

a) "kasi naumo mukuchipulikila bamama awo wakovwila vya HIV ku chipatala na kuvikaya tchito yawo nivichi kweniso mbanjani chomenemene?"

*[**PURPOSE OF QUESTION:** To determine adolescent mothers' understanding of who mentor mothers are and what they do, including key activities]*

b) Nawunkhwantha winu wa m2m/bamama wakovwila kwasintha naumo mukusangila wovyili wa vya umoyo kwa imwe na mwana winu? Chonde fotokozani viwemi panji viheni.

[PROBE]

- Kusanga unthenga wakunkhwaska za umoyo
- Kapwelerelo kakusikelo
- Kayezgelo kakwakachibungu ka HIV na uphungu
- Kutolapo lwande/Kupimika nakupokela uphungu pamoza na wakutemweka winu
- Kwamba kupokela mankhwala ghama ARV
- Uphungu wakamwelo ka makwala gha ma ARVs
- Kuwovwirika pakubaba/kuchila
- Kupimika ndopa kwa bana awo bamama ghawo wana kachibungu ka HIV
- Uphungu wa kawokheselo ka mwana
- Kubeka vya Katemela /kakulilo na thanzi la bana awo wandajum vyaka vinayi

*[**PURPOSE OF QUESTION:** To establish specific detail on which aspects of PMTCT service delivery has been changed positively or negatively because of m2m or a mentor mother]*

***Note to RAs**: It is possible (although hopefully unlikely) that participants have not experienced or perceived any changes. If this is the case, and they re-iterate that they don't think there were*

changes, do not keep probing as it is disrespectful. However, if they mention such changes in the context of another question – it is OK to probe at that point, as the participant has obviously 'made sense' of this idea in relation to a different question.]

- Kasi wovwiri wuli uli uwo mulikupokela kufuma kwa mentor mothers nakupambaniska na uwo mukupokela kufuma kwa wakugwira ntchito muchipatala?

***[PURPOSE OF QUESTION:** To establish specific examples of where adolescent mothers have been unable to access or take their ARVs and the factors contributing to these cases]*

Gawo lachinayi – wovwiri uwo mungakhumba (35 minutes)

a) "kasi niwuwovwiri wuli uwo mungatemwa kuti mupokele kuti mukhale na umoyo uwemi apo mulina nthumbo kweniso pala mukuwonkhiskanakumusunga mwana winu wambula HIV? Chifukwa wulu chifukwa wuli yayi
[PROBES]
   - Uphungu/ chovwiri cha ntchito zakupambana pambana
   - Kukumanga magulu nakuwovwiranga
   - Wovwri olo chilimbikiso chakumwa mankhwala ama ARV zubanazuba
   - Kuyowoya na wanalume wawo panji bachibale
   -
   - Mayunitsi
   - Mayendelo wakuluta naku welako kuchipatala
   - Vyakulya vakusyazilapo
   - vinyake

***[PURPOSE OF QUESTION:** To establish examples of PMTCT services adolescent mothers may want]*

"

b) "Kasi mungatemwa kuti mukhalenge na mama nge ndimwe uyo naye wanakachibungu ka HIV kuti movwiranenge nakubekisyiksa kuti mukupokela wovwiri wose wakukhumbika kuti mukhale namoyo uwemi nakuvikilira mwana binu kukachibunbu ka HIV? fotokozani pala nintheula panyake yayi."

c) "pala mungatemwa kukhala namama munyake nge ndimwe kuti wamuwovwireninge apo muli nanthumbo napala mwababa, nivinthu uli ivyo mugatemwa kuti wamupangileninge?"
[PROBES]
   - Uphungu wakamwelo ka makhwala kakwenela
   - Uphungu na kupimika ndopa nge banja
   - Katolelo ka ma ARV na mankhwala wanyake
   - Kumuyimbilani panyake kumutumilani unthenga pa foni nakumukumbuskani vya ulendo winu wakulutaso kuchipatala
   - Kwiza kunyumba kwinu nyengo nanyengo kuti tibeke umoyo binu nawa mwana pala ulimakola
   - Kupaniko vyakulya panyake wovwiri wakalyelo kawemi
   - Kumupani uphungu wa:
     - Kukhala namoyo wachigomezgo
     - Kulondezha uphungu wakuchipatala

- Kupeleka uphungu wa ivi:
  - Kuyowoya na mfumu wawo na wachibale kunkhaska HIV, nthumbo yinu
  - Umoyo wa mmalingaliro/
  - Kuchepeska kamwelo ka mowa/kugwiliska ntchito mankhala wozweteska ubongo
- Kumupani wuthenga wa masambiro/panyake mwawi wakusambirai uyo ungasangika kukaya kwinu

*[**PURPOSE OF QUESTION:** To establish specific examples of PMTCT support services adolescent mothers may want]*

d) "kasi ni vinthu mbuni ivyo vingapangiska kuti mukolelaneko nakungwirila tchito limoza makola namama munyake uyo wali na HIV kukaya kwinu na nyengo yiliyose mungaluta kuchipatala?"
[PROBES]

-

msepuka (vilimika 19 panji kuchepelapo)
bachinyamata (vilimika 20-24)
balala(vilimika 25 – 50)
mlala (opyola zaka 50)

- ivyo wakumanya
  - wawe wakusambizyika ngati wakupeleka phungu
  - wakalondezga ndondomeko ya PMTCT makola ndipo wakajisunga mwanthazi wose na mwana wawo
  -
  - utali wanyengo iyo wakharila nakachibungu ka HIV
- Malo awo mungasangana
  - Kukumana kwambula kuwonana
    - Machini ya internet panyake facebook
    - foni/uthenga/whatsApp
  - Kukumana pa malo
    - Kuchipatala
    - Kunyumba
      Ku sukulu
    - Ku malo wakusopelako
    - Kunyake
- Nyengo yakusangilana
- Utali wakhumano
- Vinyake

*[**PURPOSE OF QUESTION:** To establish specific attributes of mentor mothers and their encounters with mentor mothers that might be preferable to adolescent mothers]*

e) "Paliso vinyake vilivyonse ivyo mukhumba kuyowoyapo pambere tindajale?"
[PROBE]

- Vinyake ivyo vindadumbike

*[**PURPOSE OF QUESTION:** To provide participants with the opportunity to share any other information that may be relevant to how they access or would like to be supported for PMTCT services either now or in the future]*

Adolescent Mother Needs, Attitudes, and Preferences
Focus Group Discussion Guide

Introductory Script (follows written informed consent process):

Chonde awalanje mwakwesya: [“Aliuli. Tuyamiche kwejinji pakujigala nawo mbali mu yakuwechetana yetuyi lero jino. Lina lyangu ni ________________________ nambo timbe nikulongolela yakuwechetana yetu. Ajangu windinawi papapa lina lyakwe ni ________________________ nambo tawe anamkulemba ituwe ikwechetana apano.
Kusimanigwa kwawo yakuwechetana yalelo yitukamuchisya kuti tupate lunda mwampaka twakamuchisye achim’masyeto achinandipile ni wanache wawo kuti apiltilisye kwaula kuchipatala, kumwa mtela wa ma ARV ni kuwa ni umi wakwimbala chenene.

Yakuwechetanayi iwe ya pagulu. Ngawa iusyo ituweje kuusyana kuti ijale pa iusyo ijakwe iyayi. Itutende apano tupelecheje nganisyo kapena mtwe wa ngani wane waliwose tajigale nawo mbali pakuwechetana waliwose ni ajawo. Waliwose tuwape nambala; choonde choonde waliwose asale nambalaji pandawi jilijose tiwapw mpata kuti awechete.

Tukusaka kupikana nganisyo syagulu kapena yakusana muyakupochela kuchipatala ni mu mbali mwenu mukatama yakamuchisya kupelegana kachilombo kutyochela kwa mama kwaula kwa mwanache. Ikamuchisyoyi ikusamanyika mu lina la m’chingelesi la “PMTCT”. Soni tikusaka tupikane nganisyo zenu, kusangalala kwenu nambo soni muupangiri wenu mwapaka soni twakamuchisye mwachisimichisyo achim’masyeto amwanamwana wakwete kachilombo ka HIV kutyochela pandawi wakwete chilu mpakana palechele kuonjesya mwanache jwawo. Tikusaka kuimanya indu yakulandana nambo soni yakulekangana mugulu mwenumo. Pangali chakwanga chachilungamo kapena soni chakulepela.
Akwete ufulu kwanga iusyo naga akusaka kutenda yeleyo. Choonde choonde awe wagopoka pakuusya iusyo iliyonse naga pan ine yanganapikanichisya.

Mwagambakumbusyana, malowe tugawane apano gachisisi. Itawechete apano pangali witachimanya kuti awechete wawojo. Itujile pakuwechetana apano tuchiwanganya ni utenga wine ichityochela kuyakambilana yineyo ichipelechedwa ku wakulungwa wa zaumoyo ni mabungwe gane pakusaka kwausya pasogolo mapologalamu gakusaka kuyilambalala yakupa kachilombo kutyochela kwa achim’masyeto kwaula kwa mwanache, nambo soni pangali itachigamanya ndawi, malo kapena wandu wagulu jakambilanaji.

Akwete chiusyo chilichose nganituwe yakambilanayi?”

<u>Iusyo yakusana maganisyo komboleka kujigala nawo mbali mu pologalamu jakuteteza mwanache kuti akajigala kachilombo kutyochela kwa achim’masyeto</u>

<u>Chandanda – Utenga wandanda</u>

a) “Ana atusalile naga aicheje chiptala achino kapena chipatala chine chakumusi kandawi jelewu chinauli?
b) Ana achimanye chichi yakusana ni mother 2 mother.

*[**PURPOSE OF QUESTIONS:** Some icebreakers to establish rapport; a non-threatening introduction]*

c) Pakamulana ni indi yasimene nayo piwaliji wana msigo/wakete mwanache, ana akuganisya kuti indu yapi ikusalandana ni kwimbala kwa achim'masyeto achinandipela wakwete kachilombo ka HIV ni wanache wawo mu litala lyambone? *[Zoti mufunse]*
- Yaka/wakongwe kapena walume
- Uganja/kuwa ulombela
- Yindu yampaka yikamuchisye kuti awe an uumi wambone (masengo, kulijiganya)
- Yakulya
- Chakwendela
- Kuwa pa masengo kapena ngawa pa masengo
- Kusimanigwa kwakwe nambo soni kusalala kwa chikamuchisyo cha zaumoyoChikamuchisyo (kucjipatala nambo soni kumuzi)
- Yine

Pakamulana ni indu yasimene nayo kuwa wana msigo/mwanache, ana akuganisya kuti indu yapi ikusalandana ni kwimbala kwa achim'masyeto achinandipela wakwete kachilombo ka HIV ni wanache wawo mu litala lyakusakala? [Zoti mufusne]
- Yaka/wakongwe kapena walume
- kubenga
- Uganja/kuwa ulombela
- Yindu yampaka yikamuchisye kuti awe an uumi wakusakala (kulaga, kulijiganya)
- Yakulya
- Chakwendela
- Kuwa pa masengo kapena ngawa pa masengo
- Kusimanigwa kwakwe nambo soni kusalala kwa chikamuchisyo cha zaumoyo
- Chikamuchisyo (kuchipatala nambo soni kumuzi)
- Yine
-

*[**PURPOSE OF QUESTION:** To determine client perceptions and experiences of what factors impact their health and access to health care. NB: These could be POSITIVE or NEGATIVE factors. We are interested in both.]*

- Ana indu ayi ikusalandana ni kwimbala chenene kwa achim'masyeto achinandipela ni wanache wawo?

*[**PURPOSE OF QUESTION:** To understand HOW the issues described above are perceived to affect health]*

Chawiri –Yayimanyi yakusana nichikamuchisyo cha zaumoyo ni kamuchisya mwanache kuti akajigala kachilombo ka HIV (30 minutes)

Chonde awalanje mwakwesya: [“Sambano tukambirane yakusana chikamuchisyo cha zaumoyo ni ine yakusana kuti mwanache akajigala kachilombo ka HIV kutyochela kwa achim'masyeto kumbali jenu.”]

a) “[Ana chikamuchisyo ha zaumoyo cchikwasimana chinauli cha wawojo ni mwanache jwawo?”]

*[**PURPOSE OF QUESTION:** To establish factual detail about the processes adolescent mothers actually go through when accessing healthcare in their community. This information will help us understand the degree to which actual practice mirrors (or departs from) ideal practices (formal, recommended practices or national guidelines)]*

b) Ana mpaka atusalile mwa indi yasimene nayo pakupata chikamuchisyo chkulinjisya HIV kuchipatala chenu /kusikelo ja achim'masyeto wakwembecheya.”

[Zoti mufuse]

- Malo gambone ni gapai gampaka akalinjisye HIV nambo soni kupochela malamusi ?
- Ana akusawadalila wani kuti akalinjiesy HIV kapena kupochela malamusi?

*[**PURPOSE OF QUESTIONS:** To document adolescent mothers' experiences with HIV testing and understand adolescent mothers' perceptions of enabling factors for HIV testing]*

c) ”Ana asimene yakusausya yanti uli piwatandaga kumwa mtela wa ma ARV? Kupitlisya kumwa sooni mtela wa ma ARV? Ligongo lakwe chichi?”

Note for RAs: Awechetane nawo ni wakujigala mbali mu kafukufukuji pologalamu jakwe mujiwelele jandanda pakupochela mtela wa ma ARV kwa ndawi jandanda, muwapikanile, yakusausya yiwasimene nayo pajele ndawijo nambo soni kutyochela ndawiji pakupitilisya kumwa mtela wa ma ARV.

[oti mufunse]

a. Yaka/wakongwe kapena walume
b. kubenga
c. Uganja/kuwa ulombela
d. Yindu yampaka yikamuchisye kuti awe an uumi wakusakala (kulaga, kulijiganya)
e. Yakulya
f. Chakwendela
g. Kuwa pa masengo kapena ngawa pa masengo
h. Kusimanigwa kwakwe nambo soni kusalala kwa chikamuchisyo cha zaumoyo
i. Chikamuchisyo (kuchipatala nambo soni kumuzi)
j. Yine

*[**PURPOSE OF QUESTIONS:** To understand how adolescent mothers access ARVs and document their experience of barriers and enablers to regularly accessing ARVs]*

d) Ana asimene ni yakusausya yekulungwa uli piwalinjisyaga mwana gwawo kachilombo ka HIV? Ligongo lakwe chichi?
- Yasimene nayo paukja kulinjisya myasi nambo soni pakupochela yakuichisya ya myasi ya mwana gwawo
- Yakamuchisya kuti mwna gwawo alinjisye myasi
- Kupata mtela wa ARV wa mwana jwakupagwa kene ni mwana gwenu juli jwana msingu wam'nono

e)

f) Ana indu yapi yampaka msangalale nayo tumpanganichisye yakusana ni chikamuchisyo cha zaumoyo cha wawojo ni mwana gwawo?

- Kusimanigwa
- Ndamo sya wakamula masengo wa zaumoyo
- Mapologalamu jakamuchisya

*[**PURPOSE OF QUESTION:** To establish those factors, from the perspective of adolescent mothers, that are most important for improving access or quality of care]*

Chatatu – Yayimanyi yachim'masyeto yakamuchisya mama ni mama (35 minutes)
[SKIP if FGD is being conducted with adolescent mothers who do not currently or have not previously received m2m services.]

Chonde awalanje mwakwesya: ["Sambano ngusaka kuusya chiusyo cha kwa achim'masyeto wakamuchisya."]

a) "Ana malowe genu, mpaka wechete ana mentor mother ni wani nambo soni akuganisya kuti akukamula masengochi kuchipatala kapena ku muzi ?"

*[**PURPOSE OF QUESTION:** To determine adolescent mothers' understanding of who mentor mothers are and what they do, including key activities]*

b) "Malingana ni indu yakusiman nayo, ana m'masyetowa akamuchisye kuchenga indu yachikamuchisyo cha zaumoyo cha wawojo ni mwanache jwawo? Choonde awechete yakuchenga yambone kapena yakusakala ".

[PROBE]

- Kasimanigwe ka utenga wa za umoyo
- Kupochela chikamuchisyo cha achim'masyeto wakwete misigo
- Kwalinga myasi nambo soni kupochela malamusi
- Kwaula yalumo wakongwe ni walume/kuja kupochela malamusi nambo soni kulinijisya myasi mpela ulombela.
- Kutanda kupochela mtela wa ma ARV/wa m'masyeto wamsigo kapena wakuonjesya kupochela mtela wa ma ARV
- Malamusi wakwakamuchisya kapena kwalimbikasya pakupochela mtela wa ma ARV
- Kwika kuweleka
- Kulinga mwanache jopagwa kwene kuti panjipa akwete kachilombo ka HIV
- Malamusi gakusana ni kuonjesya kwa mwanache
  Sikelo ja wanache wangakwana yaka msanu

*[**PURPOSE OF QUESTION:** To establish specific detail on which aspects of PMTCT service delivery has been changed positively or negatively because of m2m or a mentor mother]*
***Note to RAs**: It is possible (although hopefully unlikely) that participants have not experienced or perceived any changes. If this is the case, and they re-iterate that they don't think there were changes, do not keep probing as it is disrespectful. However, if they mention such changes in the context of another question – it is OK to probe at that point, as the participant has obviously 'made sense' of this idea in relation to a different question.]*

c) Ana chikamuchisyo chapochele kwa mentor mothers ikulekangana uli chapochele kuchipatala kapena wakamula masengo wa za umoyo?

*[**PURPOSE OF QUESTION:** To establish specific examples of where adolescent mothers have been unable to access or take their ARVs and the factors contributing to these cases]*

Gawo ja mcheche – Chikamuchisyo cha mpaka achisose (35 minutes)

a) "Ana muchikamuchisyo chichi cha mpaka achinonyere pukutama pa umi wao pali wakwembecheya soni wakuonjesya ni kukamuchisya mwanache jwenu kuti akajigala kachilombo ka HIV? Ligongo chichi ili yoyo ni ligongo lyakwe chichi ili soni iyoyo"
[PROBES]
   - u m'miziMalamusi/masengo gakamuchisya wand
   - Wandu wakamuchisya magulumagulu/kuwechetana kwa mundu jwine ni jwine
   - Kukumbusya mundu kuti amwe mtela wa ma ARV lisiku ni lisiku
   - Mayunitsi ga lamya
   - Thalansipoti jakutyochela ku nyumba mpakana soni kuchipatala
   - Yakulya yakuonjechesya
   - Yine yakuonjechesya

***[PURPOSE OF QUESTION:** To establish examples of PMTCT services adolescent mothers may want and why they may be wanted]*

b) Ana mpaka msose wa m'masyeto wanondi wakwete ka chilombo ka HIV wakuti akamuchisyeje pakulolechesya kuti mukupochela chikamuchisyo chosope pakuwa muntendele ni kunka muchisya mwana gwenu kuti akajigala kachilombo ka HIV. Atusalile naga iri yoyo."

c) Ana mdili sagwile kuwa ndi wa m'masyeto wam'nono wakwete kachilombo ka HIV kuti ankamuchisyeje pimdili pakakati pakwembechela nambo soni dimbeleche mwanache, chikamuchisyo chichi champaka mchisose kuti amkamuchisyeje wa m'masyeto ajenu

[PROBES]
   - Uchimbichimbi ni kusosegwa kumwa kwa mtela kwambone
   - Uchimbichimbi ni kusosegwa kulinjisya kwa myasi kwa ulombela wosope
   - Kuchigalidwa kwa ma ARV ni mitela jine
   - Kwajimbila telefoni kapena kwatuchisya utenga kwakumbusya masiku gakusosegwa kuti akawe kuchipatala
   - Kwajendela kunyumba jawo ndawi ni katema kuti twalole mwawelele wawojo ni mwana gwawo
   - Kwapa kaposo ka yakulya kuti awe wana chikamuchisyo chakwanila mchilu mwawo kapana chikamuchisyo cha pakulya chokupeleka kwimbala mchilu mwawo
   - Kwapa uchimbichimbi wa:
     - Kutama umi wakwembecheya
     - Kuya upangiri wakuchipatala
   - Kwapa uchimbichimbi wa wa izi:
     - Mwampaka awechetele wankwenu kapena a pa mtima penu yagundana ni kachilombo ka HIV ni chilu chenu
     - Uumi waganisyaganisya
     - Kunandiya kumwa kwa ukana/kunandiya soni kumwa indu yakulekanganalekangana yakusungusya mtwe
     - Kwapa utenga wakulijiganya/kapena upile wakulijiganya wampaka upatikane kumangwenu
     - Yine

*[**PURPOSE OF QUESTION:** To establish specific examples of PMTCT support services adolescent mothers may want]*

d) "Ana indu yapi yampaka iwe yakomboleka yakuti mbeyalumo ni achim'masyeto wakwete kachilombo ka HIV pakamula masengo ya lumo ku muzi kwenu nambo soni ndawi jilijose jimpite kuchipatala?"

[PROBES]

- Yaka ya mundu
  -
  - Wachinandipile (yaka 19 kapena kunondipila pelepo)
  - Jwelejo Mnyamata (yaka-20 mpaka 24)
  - Wankulungwa(yaka 25 – 50)
  - Wankulungwa mnope (wakupundana yaka 50)
- kumanyilila chenene kwa mayi wakamuchisya
  - Awe wakulijiganya
  - Wapochele chikamuchisyo cha PMTCT nambo soni wele mwanjawo ni mwana gwawo waji wakwimbala pampepe ni mwana gwawo
  - Kutalika kwa yaka yatemi ni kachilombo ka HIV
- Malo gampaka msimane
  - Kusimana mwangalolegana
    - Pana isyano ya pa makinan ga internet kapena soni facebook
    - Telefoni/utenga/whatsApp
  - Kusimana pa malo
    - Kuchipatala
    - Kunyumba
      Ku sukulu
    - Ku malo gakuswalila
    - Yine yakusosegwa
- Ndawi jakusimanilana
- Kuutalika kwa ndawi jakuoneganilana
- Yine

*[**PURPOSE OF QUESTION:** To establish specific attributes of mentor mothers and their encounters with mentor mothers that might be preferable to adolescent mothers]*

e) "Pana chilichose chakusaka kusala nganituwe kumalisya?"

[PROBE]

- Chine chilichose chinganawecheta

*[**PURPOSE OF QUESTION:** To provide participants with the opportunity to share any other information that may be relevant to how they access or would like to be supported for PMTCT services either now or in the future]*
